# Phage therapy for environmental biotechnology applications

**DOI:** 10.3389/fmicb.2025.1621103

**Published:** 2025-09-03

**Authors:** Suniti Singh, Rachel Samson, Francis Hassard

**Affiliations:** ^1^Cranfield University, Bedfordshire, United Kingdom; ^2^College of Science, Engineering and Technology, Institute for Nanotechnology and Water Sustainability, University of South Africa, Johannesburg, South Africa

**Keywords:** phage therapy, patent landscape, soil-vegetable system, biofuel system, engineered water system

## Abstract

Environmental compartments, from soils and crop rhizospheres, to bio-reactors and municipal water networks have emerged as dynamic hot-spots for antimicrobial-resistance evolution and dissemination. Bacteriophages offer a precision, self-amplifying alternative to conventional biocides, yet their environmental deployment, intellectual-property space and commercial readiness remain only partially charted. Here, we critically synthesize the past decade of progress in phage-based interventions across three sectors: (i) soil remediation and crop-protection interfaces, where multi-phage cocktails suppress wilt- and blight-causing pathogens while preserving beneficial microbiota; (ii) biofuel and petro-energy infrastructures, in which lytic phages mitigate the microbiologically influenced corrosion and contaminated fermentations, restoring ethanol yields; and (iii) natural and engineered water systems, where phages show promise in treating recalcitrant biofilms, algal blooms and selectively ablate World Health Organization-priority pathogens. Meta-analysis of the World Intellectual Property Organization database reveals rapidly rising but geographically skewed patent activity, with China and the United States accounting for >61% of reviewed filings, and a gap between laboratory proof-of-concepts and marketed products. We identify bottlenecks, including lack of good manufacturing practice at scale, fragmented regulatory frameworks, and the evolutionary balance between single-phage precision and cocktail breadth. A roadmap is suggested that couples high-throughput phage discovery, synthetic tailoring and adaptive approval pathways. Together, these advances position environmental phage therapy to become a cornerstone of the One-Health response to increasing levels of microbial resistance.

## Graphical abstract

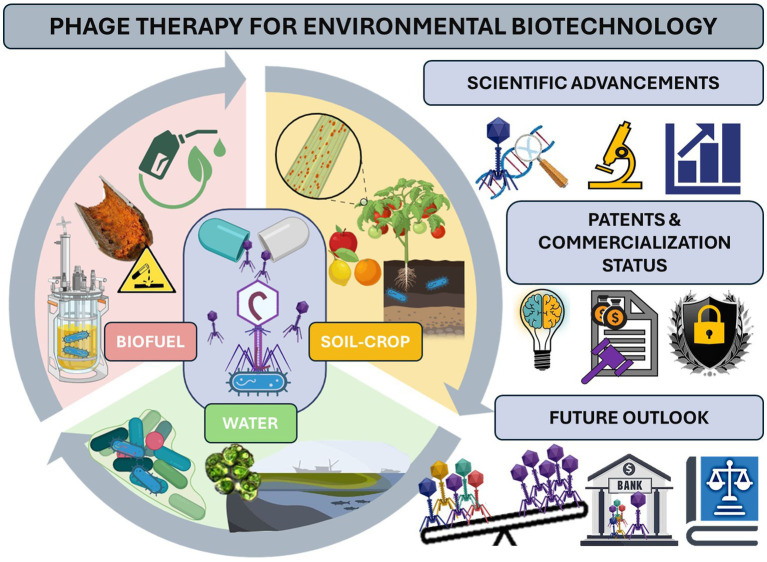

## 1 Introduction to phage therapy

Phage therapy harnesses obligately lytic bacteriophages (viruses of bacteria) to precisely lyse pathogenic bacteria without impacting non target cells. Therapeutic phage candidates should demonstrate high infectivity and host specificity, clear genomic safety (no toxin, lysogeny, or antimicrobial-resistance (AMR) genes), environmental stability, formulation compatibility, and amenability to scalable manufacturing. Once a niche practice, phage therapy is now growing as the AMR challenge intensifies. The global phage-therapy market is projected to reach ≈ USD 1.65 billion by 2030 (Compound Annual Growth Rate (CAGR) ~ 4%) (Coherent Market Insights, 2025; Mordor Intelligence, 2025). North America currently holds the largest share, but Asia–Pacific is poised for the fastest expansion ~7% CAGR as biotechnology investment accelerates through to 2028.

The field’s momentum aligns with the One-Health paradigm, which recognizes the interconnectedness of human, animal, and ecosystem health. Environmental compartments act as reservoirs of resistance: antibiotic residues and resistant microbes enter soils and waters via agricultural runoff, wastewater, and pharmaceutical waste, sustaining selection and horizontal gene flow. These genes feed back into human and animal populations. AMR as attributed to 1.27 million deaths and contributed to 4.95 million more in 2019, with economic projections surpassing USD 3.4 trillion in lost GDP and USD 1 trillion in extra healthcare costs by 2050 (Naghavi et al., 2024) if spread is not mitigated. Controlling bacterial loads in these non-clinical spaces is therefore needed to disrupt environmental feedback loops that accelerate AMR dissemination.

Literature reviews on phage therapy applications in recent years have predominantly focused on clinical practices addressing human health, animal health, plant diseases, agriculture and aquaculture, targeting specific pathogenic bacteria, while a comprehensive analysis in the environmental matrices remains limited (Strathdee et al., 2023; Haq et al., 2024). Given the significant role the environmental matrices play as reservoirs for AMR, coupled with their substantial financial implications, a focused discussion on this topic is needed. To study the phage technology development, we also review a decade of World-Intellectual-Property-Organization filings and commercial launches, revealing sharp geographic asymmetries and an enduring bench-to-market gap. Because kilolitre-scale, good manufacturing practices (GMP)-compatible manufacturing and harmonized approval pathways remain major rate-limiting steps, we synthesize recent progress in bioprocess intensification and discuss emerging “master-bank” or “phage-library” regulatory models that could streamline adaptive cocktails. Here, we review recent advances in three domains: (i) soil remediation and crop–soil interfaces, (ii) biofuel and petro-energy production, and (iii) natural and engineered water systems. We further highlight synthetic-biology tools from receptor-binding-protein engineering and directed evolution to recombinant endolysins that are widening host range and opening new industrial frontiers, and we frame the evolutionary tug-of-war between single-phage precision and cocktail breadth as a central formulation dilemma. We aim to provide a concise cross-sector roadmap for deploying phage biotechnologies as an integral part of the global One-Health response to AMR.

## 2 Current research of phage therapy in environmental systems

### 2.1 Soil remediation and soil-vegetable systems

Phage therapy has gained rapid traction as a biocontrol strategy in soils and soil–vegetable interfaces, providing a precision, self-amplifying alternative to copper bactericides and antibiotics for pathogens such as *Pseudomonas syringae*, *Xanthomonas* spp. and *Ralstonia solanacearum* (Bisen et al., 2024). High-throughput isolation coupled with long-read genomics was used to certify absence of lysogeny or AMR genes. This has yielded “phage banks,” and three phage active ingredients (marketed as AgriPhage^™^) which have cleared U. S. Environmental Protection Agency (USEPA) Microbial Pesticide registration (USEPA, 2011). A separate EPA decision approved phage active-ingredients against *Erwinia amylovora* (EPA, 2018).

Greenhouse and field assays show that individual phage strains (e.g., ɸsp1) have achieved ~82–88% wilt reduction in tomato seedling assays (Wang et al., 2020). Commercial phage formulations have advanced to orchard-scale applications; however, UV exposure promotes pyrimidine dimerization in DNA, thereby limiting phage persistence. To address this, evening sprays and phage formulations containing UV-shielding adjuvants (such as peptides, aromatic amino acids, polysorbate, kaolin, pregelatinized corn flour, sucrose, skim milk formulations, or pigments like carotenoids and betalaines) have been employed to extend the phage activity sufficiently to overlap with pathogen infection windows (Born et al., 2015; Ke et al., 2024). These findings underline the need for next-generation UV-protective formulations or evening spraying regimes to ensure field trials are successful. Beyond direct pathogen inactivation, metagenomic surveys indicate that soil phages modulate nutrient cycling and contribute to auxiliary metabolic genes that improve host fitness (Wang et al., 2024a). These agronomic and ecological gains position bacteriophages as an emerging component of sustainable agriculture, advancing One-Health objectives by limiting environmental AMR reservoirs while enhancing soil-ecosystem function.

### 2.2 Biofuel and petro-energy production systems

Fuel-ethanol fermentations are commonly run under non-sterile conditions because full steam-sterilization of 100–250 m^3^ vessels would impose an energy penalty. This compromise invites chronic contamination, wherein next-generation sequencing surveys show that lactic-acid bacteria (LAB) dominate the microbial load. A single bloom of *Lactobacillus fermentum* can cut ethanol yields by up to 27% and trigger costly shutdowns (Bischoff et al., 2009). Historically, plants have dosed oxidizing chemicals or antibiotics such as virginiamycin; however, *Lactobacillus* isolates from medicated facilities carry the *vat(E)* efflux gene (Khatibi et al., 2014), and concerns about antibiotic use are posited due to residues persisting in distillers’ grains. A study showed that phage cocktails can recover ethanol yield in *L. fermentum*–contaminated fermentations to near control level (Liu et al., 2015); pilot-scale deployments are needed to validate yield recovery at equivalent dosing levels with no adverse effects on yeast viability.

While phage cocktails demonstrate strong standalone performance in recovering ethanol yields, integrating complementary agents such as phage-derived endolysins could further stabilize fermentation processes. Wang et al. (2020) reported recovering ~3.3 g/L of thermostable lysin (TSPphg) from a 20 L *E. coli* fed-batch, a value which is the current maximum in microbial hosts (Cremelie et al., 2024).

### 2.3 Natural and engineered water systems

The persistence of hard-to-eradicate microbes including water-borne pathogens, bloom-forming cyanobacteria, filamentous “foaming” bacteria and recalcitrant biofilms, poses an escalating challenge for aquatic systems worldwide. Outbreaks of *Vibrio cholerae*, *Escherichia coli*, *Legionella* spp., *Leptospira* and *Salmonella enterica* serovar Typhi are showing geographically shifting incidence patterns tied to environmental change. Since 2010, the Global Task Force on Cholera Control has recorded millions of suspected cholera cases and tens of thousands of deaths, with South Asia bearing the heaviest burden. The WHO priority list 2024 named 15 antibiotic-resistant bacteria for accelerated R&D, ranking water-associated carbapenem-resistant *Acinetobacter baumannii* and third-generation-cephalosporin/carbapenem-resistant *Enterobacterales* as “critical” (WHO Bacterial Priority Pathogens List, 2024).

Target-specific phages have been isolated from rivers, wastewater and sewage for many of these pathogens, including *E. coli*, *A. baumannii*, *Shigella*, *V. cholerae*, *Salmonella*, *Citrobacter freundii*, *Pseudomonas aeruginosa* and *Klebsiella*, and repeatedly reduce bacterial loads in laboratory and pilot studies (Ji et al., 2021). WHO conducted earliest trials of anticholera phages *in vivo*, and demonstrated that seasonal cholera epidemics in Bangladesh are modulated by rising environmental vibriophage levels, leading to self-limitation (Marcuk et al., 1971). Anticholera phages lowered *in-vivo V. cholerae* counts, and long-term environmental monitoring in Bangladesh revealed self-limiting cholera waves as vibriophage titres rose (Faruque et al., 2005). A three-phage cocktail (pSf-1, pSb-1, pSs-1) suppressed *Shigella* in animal model (Jun et al., 2016). Although no lytic phage for *Legionella* has yet been recovered, a study identified CRISPR spacer matches to *Microviridae*-related sequences in *Legionella*, indicating yet-to-be-isolated phages (Deecker et al., 2021).

Cyanobacterial blooms create multimillion-dollar annual burdens. While strain-specific cyanophages for *Microcystis* are known (Zhang et al., 2022), field deployment is nascent: a Lake Baroon trial achieved 95% cell lysis but regrowth ensued as resistance emerged (Tucker and Pollard, 2005). Infection of *Aphanizomenon flos-aquae* by cyanophage vB_AphaS-CL131 demonstrated that phage infection alters ^15^N_2_ assimilation and thus algae nitrogen metabolism. While these findings underscore the potential of phages for bloom control, they also highlight two critical challenges: the rapid emergence of phage resistance under field conditions, and the need for careful evaluation of ecosystem-level effects following viral-mediated lysis events. Future deployments should integrate adaptive phage strategies and longitudinal environmental monitoring to mitigate unintended consequences with minimal ecosystem disruption (Kuznecova et al., 2020). Lytic phages from *Myoviridae* family such as SN-phage, GTE7 and SPI1 can suppress foaming filamentous bacteria in activated sludge (Vesga-Baron et al., 2022), but no phage has yet been isolated for the ubiquitous foam forming organism—*Microthrix* (Batinovic et al., 2019). Bhattacharjee et al. (2015) reported that membrane flux decreased from ~15 to ~47 L/h·m^2^ due to *Delftia tsuruhatensis* biofilm formation, but the application of phage DTP1 restored membrane flux to 70% of unfouled baseline. Ayyaru et al. (2018) demonstrate that *E. coli* phage P2 significantly reduce the antibiotic-resistant biofilms on PVDF–graphene membranes. Zhang et al. (2024) show via metagenomics that viral predation in anaerobic digestion predominantly reduces antibiotic resistant bacteria loads, with limited horizontal gene transfer of antimicrobial resistance genes (Zhang et al., 2024). Environmental phage applications are a promising strategy for aquatic pathogen control and bottom-up control of engineered processes.

## 3 Patent landscape and commercialization status of phage therapy in environmental systems

Patent data offer a forward-looking lens on where laboratory breakthroughs are moving toward market (van Rijn and Timmis, 2023). A World Intellectual Property Organization (WIPO) search covering January 2016 – April, 2025 returned 382 raw applications relating to environmental phages. After de-duplication at the simple-family level and manual relevance checks (search logic in Supplementary Table S1), 26 unique patent families remained (≈ 19% of hits): 11 for soil applications, 10 for bio-fuel/oil systems and 5 for water treatment were obtained (Table 1).

**Table 1:** Summary of patent applications (2016–2025) from WIPO, including key novelty and classification details, categorized under soil remediation and soil–vegetable systems, biofuel systems, and natural and engineered water systems.

Category	Sub-category	Title	Country	Application ID	I P C	Novelty
Soil remediation and soil-vegetable system	Antibiotic resistance and soil contamination control	Phage and use thereof in soil remediation	US	US289037337	B09C 1/10; C12N 7/00	Synergistic use of biochar and φYSZPK phage to suppress antibiotic resistance spread in soil-vegetable systems
		Phage composition and use thereof in inactivating antibiotic resistance pathogenic bacteria	US	US289038762	B09C 1/10; C12N 7/00	Targeted inactivation of antibiotic-resistant bacteria using mixture of 3 phages by application to contaminated soil–plant system
		Biomass charcoal-bacteriophage combined remediation method for soil-vegetable system treatment	CN	CN428095688	B09C 1/10; B09C 1/00	Integrated biomass charcoal and phage protocol for improved contact by introducing external multivalent bacteriophages
		Soil remediation agent containing multivalent bacteriophage for polluted farmland treatment and preparation method of soil remediation agent	CN	CN401642144	C09K 17/40; C12N 7/00; C12N 7/02; A01N 63/40; A01P 1/00; C12R 1/92; C09K 101/00	Formulation of a complex phage-based soil repair agent combining nutrients and phage strains for farmland detox
		Polluted soil remediation equipment and remediation method based on phage therapy	CN	CN401636078	B09C 1/10	Automated system combining a bacteriophage preparation device and a dosing mechanism for in-situ treatment of polluted soil
	Soil fertility and ecological enhancement	Method for evaluating biosecurity of deep sea bacteriophage by utilizing soil flora	CN	CN322595440	C12Q 1/6869; C12Q 1/26; C12Q 1/34; C12Q 1/28; C12Q 1/04; G01N 21/31	Use of deep sea bacteriophages and 16S rRNA sequencing to assess biological safety issue by monitoring changes in soil ecological functions in terresterial soils
		Phage and application thereof in compensation of soil nitrogen fixation	CN	CN438249064	C12N 7/00; A01B 79/00	Phage-mediated nitrogen fixation by Klebsiella phage YSZKA to reduce cost and overcome competition of traditional microbes
		Phage composition and application thereof in strengthening soil carbon sequestration	CN	CN437950485	C12N 7/00; A01B 79/00; A01B 79/02; C09K 17/14; C09K 17/32; C09K 101/00; C12R 1/92	Phage mixture constituting multivalent bacteriophages phi YSZKP, bacteriophage [phi] YSZBA1, and phage phi YSZBA2 added to soil to promote the host carbon fixation process through auxiliary metabolic genes injection
	Disease control in crops	Storage device for collecting bacteriophages in soil and crops	CN	CN412771880	B65D 25/10; B65D 25/02	Shock-absorbing protective box for phage test tubes with a storage block isolated from direct impact, ensuring reduced friction and safe retrieval
		Lysing bacteriophage and application thereof in prevention and control of tobacco soil-borne bacterial wilt	CN	CN325817805	C12N 7/00; A01N 63/40; A01P 1/00; C12N 1/20; A01G 22/45; A01G 7/06; C12R 1/92; C12R 1/01	Application of lytic bacteriophage FQ44 to control tobacco bacterial wilt by targeting *Ralstonia solanacearum* via root irrigation at optimal MOI of 1–10
		Method for improving biocontrol bacteria to prevent and control soil-borne bacterial wilt by using obligate bacteriophage	CN	CN391819471	C12N 1/20; C12N 7/00; A01N 63/40; A01N 63/20; A01P 1/00; A01G 13/00; A01G 22/05; C12R 1/01	Combination of evolved Ralstonia-resistant biocontrol strain YL-Ste-01 evo and lytic phage YL-Ste-01P to suppress tomato wilt with enhanced antibacterial effect
Biofuel production systems	MIC control and biocidal compositions	Bacteriophage mediated biocontrol in oil reservoirs	IN	IN300871536	C12N 7/00	Thermostable phage cocktail effective at 45–55 °C for use in oil reservoirs
		Bacteriophage mediated biocontrol in oil reservoirs	WO	WO2020152720	C12N 7/00; C09K 8/00; C02F 3/34; C09K 8/54	Bacteriophage cocktail targeting SRB and facultative anaerobes, stabilized with alginate, chitosan, silver, or selenium nanoparticles at 45–55 °C
		Bioassisted treatment of microbiologically influenced corrosion in petroleum transporting pipelines	US	US339381900	C23F 15/00; C09K 8/54; C12Q 1/24; C12N 11/02; B82Y 5/00; B82Y 40/00	Microbicidal composition comprising a bacteriophage immobilized on a magnetic nanocomposite, and phage releasing reagent to mitigate MIC in internal surface of the oil transporting pipelines
		Directional adjustment and control method for oil reservoir endogenous functional microbes	CN	CN295317861	E21B 43/22; E21B 43/20; E21B 47/00; C09K 8/582	Field-ready control method using SRB-specific phages and activator system to enhance oil recovery efficiency by >20% and reduce the costs by >30%
		Phage, bactericidal composition and application	CN	CN399877696	C12N 7/00; A01N 63/40; A01P 1/00	Broad pH range (2–12), high thermal resistance (70 °C, 60 min), and low chloroform sensitive bacteriophage for targeting metal corrosion caused by SRB
		Altering microbial populations and modifying microbiota	EP	EP341188275	C12N 9/16; A01N 63/00; A61K 31/7105; C12N 15/113	Engineered sequences and vectors to alter bacterial subpopulation ratios or inhibit specific microbes in MIC or biofouling
		Inhibition of bacterial growth in pipelines	US	US254126853	F16L 58/00; A01N 59/00; B08B 9/027; C02F 3/34; C09K 8/54; B08B 9/055; B08B 17/00	Use of freshwater-induced osmotic shock combined with biocides and pigging for killing the bacteria responsible for reservoir souring
		Treating and preventing microbial infections	WO	WO2019185551	A61K 38/46; C12N 9/22; C12N 15/113; A61P 31/00; A61P 31/04	Phage-delivered programmable nuclease for controlling MIC and biofouling
		Altering microbial populations and modifying microbiota	WO	WO2016177682	A01N 63/00; A61K 31/7105; C12N 9/16; C12N 15/113	Phage-delivered engineered nucleotide sequences for controlling MIC and biofouling.
	Fuel cell and power generation	Activity regulator of current-generating bacterium, and output regulating method of microbial fuel cell system	JP	JP283247419	C12N 7/00; C12N 1/20; C12Q 1/70; G01N 27/327; H01M 4/90; H01M 8/0485; H01M 8/08; H01M 8/16	Use of bacteriophages to modulate current-generating bacterial population to control extracellular electron transport mechanism and regulate output current in microbial fuel cells without chemical mediators, enhancing power generation efficiency
Natural and engineered water systems	Biofilm system	Consortium of Broad-spectrum jumbo bacteriophages for control of multidrug resistant *Pseudomonas aeruginosa*	IN	IN377285493	A61K /; C12N /; C07K /; C07K /; A61P /	Broad-spectrum jumbo bacteriophage consortium targeting biofilm-forming multidrug-resistant *P. aeruginosa*
	Algal biocontrol	Bacteriophages with improved antimicrobial activity	WO	WO2023015195	A61K 35/76; A61K 38/51; C12N 9/88; A61P 31/04	Natural phage-based water disinfectant mimicking River Ganges purification mechanism
		Bacteriophages with improved antimicrobial activity	US	US441476285	C12N 7/00; C12N 9/22; C12N 9/88; C12N 15/11; C12N 15/90	Genetically engineered bacteriophages expressing alginate lyase EPS depolymerase for infection treatment
		Biocontrol compositions of bacteriophage, methods of producing and uses thereof	WO	WO2024091978	A61K 35/76; A61P 31/04; C12N 7/02	Genetically engineered bacteriophages expressing alginate lyase EPS depolymerase for infection treatment
	Water disinfection	A novel approach to prevent contamination of domestic water using bacteriophage as biological-disinfectant	IN	IN362988518	A61K /; C12N /; C12Q /; G01N /; C07K /	Lytic phage-based biocontrol formulation effective against a broad range of pathogenic *E. coli*

Soil-sector filings rose from one in 2016 to four in 2024, a steady upward trend (≈ 19% CAGR). Four International Patent Classes (IPC) classes dominate: C12N 7/00 (bacteriophages), B09C 1/10 (soil bioremediation), A01N 63/40 (biocontrol agents) and C09K 17/40 (soil conditioners). China accounts for ≈ 42% of families, while the United States for ≈ 19%. Patent claims cluster around (i) suppression of antibiotic-resistant bacteria in manure-impacted soils, (ii) phage-assisted nitrogen fixation or carbon sequestration and (iii) cocktails for control of bacterial-wilt and fire-blight pathogens.

For bio-fuel, oil and corrosion, 10 families were filed, most after 2020, reflecting a recent increase in interest toward microbiologically influenced corrosion (MIC) control and enhanced oil recovery (EOR) (Table 1). Applicants range from start-ups (Gangagen Biotech) to national oil companies (Oil India Ltd) and majors such as Sinopec. IPC codes center on C12N, A01N/C09K (biocides, anticorrosives) and H01M (fuel-cell microbiomes).

Water-treatment patent filings remain sparse: only five patent families survived screening (two PCT, two Indian, one US), of which three from private entities (Table 1). Assignees include Armata Pharmaceuticals, SUG Biosciences and Indian Oil Corporation. Claims span phage cartridges for RO-membrane biofilm removal, cyanophage concentrates for bloom control and alginate beads for potable-water disinfection, filed mainly under C12N and A61K. The narrow pipeline underscores the technical and regulatory hurdles still facing large-scale phage deployment in chemically variable water matrices.

Despite the 26 unique active families identified in this review, commercial roll-out is modest. AgriPhage™ (Phagelux, China) foliar sprays for fire blight, bacterial wilt and other crop diseases reported low-single-digit-million-USD domestic sales in 2023 (company press release, 2024) and remains the best-selling environmental phage product. Intralytix (United States) leads the field with multiple EPA-registered phage formulations, such as ListShield (for *Listeria*) and EcoShield (for *E. coli*) areapproved for environmental decontamination in food-processing and related industrial settings. A handful of research institutes and small biotech firms offer contract phage isolation and on-demand cocktail formulation services for wastewater and biofilm problems. However, these remain bespoke or limited-run solutions, and there are currently no widely distributed, off-the-shelf phage products targeting ethanol fermenters, crude-oil pipelines, or municipal scale waterworks, to the best of the authors knowledge.

The translation gap stems from three factors:

1. Scarce field-scale efficacy data that benchmark phages against incumbent chemicals;
2. Cost and regulatory ambiguity surrounding GMP-level cocktail production;
3. Fragmented national and international regulatory oversight.

Co-ordinated pilot trials, ISO/GMP production templates and sector-specific approval routes framed within a One-Health narrative could narrow this gap. Targeting high-margin sectors is critical for the commercial viability of environmental phage technologies, given persistent challenges in production scalability, regulatory complexity, and competition from established chemical controls. Applications with clear unmet needs and tolerance for premium interventions should be prioritized, including: (i) high-value specialty agricultural systems (e.g., organic cultivars, controlled-environment agriculture, precision rhizosphere control); (ii) MIC control in critical pipeline infrastructure, where conventional biocides fail or face usage constraints and partial efficacy justifies use in high-risk assets; (iii) contamination management in high-value specialty fermentations (e.g., bioethanol, specialty biochemicals) to prevent batch failures, safeguard yields, and enable cost recovery despite premium pricing; and (iv) outbreak control in acute contamination scenarios, especially for resistant pathogens in biofilms or distribution systems, where phages complement existing disinfection during high-risk events. Strategic deployment in these sectors may bridge the gap between patent activity and market uptake, supporting regulatory investment and production scale-up. Given current filing momentum and early regulatory moves, the first commercial phage anticorrosion additive is likely to reach market within the next 5 years.

## 4 Challenges and future outlook in environmental systems

### 4.1 Host specificity: molecular determinants and operational implications

Host-range precision is both a strength and weakness in phage therapy for environmental biotechnology applications. Surveys show that most lytic phages infect only a few tested strains. In a survey of *Klebsiella* phages across 138 strains, 42 of 46 phages (≥91%) infected three or fewer strains, averaging under 2% host coverage per phage (Beamud et al., 2023). This implies a key challenge for phage therapy in safeguarding beneficial native microbiota while limiting environmental dispersal within genetically diverse and spatially dynamic bacterial communities (de Jonge et al., 2019). Infection begins when virion adhesins dock onto envelope receptors (O-antigen side-chains, core LPS, wall-teichoic acids, outer-membrane porins, type IV pili or flagella) the molecular “locks” for the phage “key” (Broeker and Barbirz, 2017; Venturini et al., 2022). This is because these structures diversify rapidly, most phages recognize only a narrow phylogenetic subset (Beamud et al., 2023). Such selectivity is valuable in the rhizosphere or activated sludge, where functional components of the microbiome, e.g., nitrifiers and plant-growth promoters should be avoided. For example, in *Salmonella* phage panels, individual phages often infect only one or a few serovars; a monophage for PT4 fails against PT8 or co-resident *Klebsiella* strains, illustrating incomplete control (Beamud et al., 2023; Wang et al., 2024b). Studies where broader host range has been engineered via tail-fiber swaps report effective range expansion but phages pay a ‘fitness cost’ due to reduced adsorption rates and burst size (Dunne et al., 2019). Each engineered lineage, however, is a new biological entity with its own safety dossier and regulatory path (Samson et al., 2024). *In-silico* tools, e.g., PHERI, vHULK predict the probable hosts directly from metagenomic contigs (Amgarten et al., 2022; Baláž et al., 2023). This effectively reduces the wet-lab screening burden and accelerating targeted phage discovery from complex environmental samples.

Rational phage cocktails mitigate these limits. High-throughput screening workflows against broad isolate libraries, plus whole-genome sequencing to exclude lysogeny, toxins and ARGs, yields complementary candidates (Molina et al., 2022). Phages are amplified and produced at ≥10^7^–10^8^ PFU mL^−1^ per phage, then concentrated/filtered (e.g., ultracentrifugation) and mixed in defined ratios (Jończyk-Matysiak et al., 2021). Most environmental formulations contain single digit numbers of phages, balancing coverage with manufacturing complexity and maintain quality control feasibility (Holtappels et al., 2021). Evolution experiments illustrate the payoff, for example, Martinez-Soto et al. report a five-phage Salmonella cocktail yielding a bacteriophage-insensitive-mutant frequency of 6.22 × 10^−6^ PFU mL^−1^ (much lower than with single phages) over their assays four-phage cocktail targeting O-antigen, porins, flagella and an efflux pump (Martinez-Soto et al., 2024). When resistance finally arises, it often carries fitness costs including truncated LPS or lost motility. Multiple studies show that phage-resistant mutants often bear LPS-structure deletions or motility defects, reducing *in vivo* fitness and sometimes increasing antibiotic sensitivity (Liu et al., 2015).

Host specificity remains both the strength and the bottleneck of environmental phage therapy; genomics-guided cocktail design, machine-learning host prediction and receptor engineering together offer the most pragmatic route to broaden coverage while preserving ecological precision. Ultimately, balancing host-range precision with breadth of coverage is not merely a formulation challenge, but an important ecological consideration for environmental application of phages therapy.

### 4.2 Scaling production of phages

Industrial-scale phage manufacture now integrates biopharmaceutical–style upstream intensification with vaccine-grade downstream polishing to deliver well-defined, GMP-compliant APIs. Upstream, high-cell-density fed-batch and continuous stirred-tank (CSTR) cultivations routinely achieve high titres up to 10^11^ PFU/mL, orders of magnitude above traditional batch fermentations (Mancuso, 2020). Many facilities employ single-use, closed bioreactors equipped with in-line optical-density for real-time monitoring of host growth and phage production (Matthew, 2022), and capacitance sensors are used widely in mammalian studies and could be deployed for phages. Emerging intensification strategies, such as perfusion or alternating tangential-flow reactors which are used in vaccine/viral vector contexts could boost productivity while maintaining tight process control, but no published phage-specific data are available.

Downstream, workflows mirror those used for viral vaccines: primary clarification by depth filtration followed by tangential-flow ultrafiltration (100–300 kDa MWCO) to concentrate phages and reduce endotoxin by 1–2 logs. Rebula et al. (2023) showed that an 8 mL CIMmultus OH capture step removed 98% of host proteins and >99% of host DNA with 100% phage recovery. A follow-up polishing step on 8 mL CIMmultus H-Bond or PrimaS columns achieved a 7 log₁₀ reduction in endotoxin while maintaining phage infectivity, as confirmed by HPLC analytics correlated to drop-assay titration. CIMmultus® monoliths also support very high virus binding capacities up to 6 × 10^13^ PFU/mL have been demonstrated, enabling rapid, high-throughput viral purification. Process Analytical Technology tools such as digital PCR for identity/quantification and inline Limulus Amoebocyte Lysate assays for endotoxin are being piloted to accelerate real-time release testing in broader biomanufacturing but are recommended for phage specific production. These tools specifically detect lipopolysaccharide which is the “endotoxin” carried in the outer membrane of Gram-negative bacteria, and is not triggered by viral proteins or nucleic acids.

Validated GMP processes aim for low residuals (<5 EU/mL endotoxin, <10 ng/mL host DNA) based on vaccine monographs, although phage-specific regulatory guidelines have yet to fully evolve. Together, developments in upstream and downstream advances—coupled with genomic safety screening and real-time analytics provide a framework for scalable, cost-effective production of environmental phage products under emerging GMP frameworks.

### 4.3 Regulatory challenges and opportunities for environmental phage applications

Regulatory oversight of bacteriophage-based interventions is now the principal rate-limiting step in translating laboratory successes into routine environmental practice. In the therapeutic arena the pathway is well defined: in the United States, any phage intended to treat or prevent disease is classified as a “biological product” and reviewed by the FDA’s Center for Biologics Evaluation and Research under the IND/GMP rubric used for vaccines and monoclonal antibodies (Plaut and Stibitz, 2021). Although no product has yet reached full licensure, several first-in-human and emergency protocols are active; the 2022 IND for Adaptive Phage Therapeutics’ PhageBank which evaluated a dynamic quality-controlled phage library and matching algorithm rather than a static cocktail, represents a critical regulatory milestone. The European Medicines Agency takes the same position: phages are medicinal products, and its 2023 veterinary guideline (EMA, 2023a) formally adopted as agency policy (EMA, 2023b). This requires a traceable master bank, fully annotated genomes free of lysogeny, toxins and AMR genes, plus potency assays demonstrating each phage’s contribution to spectrum and resistance management. Both regulators are piloting “phage-bank” or master-file schemes that would allow a pre-vetted phage to be swapped into a licensed cocktail without reopening the entire regulatory dossier (Lea-Smith et al., 2025).

**Figure 1:**
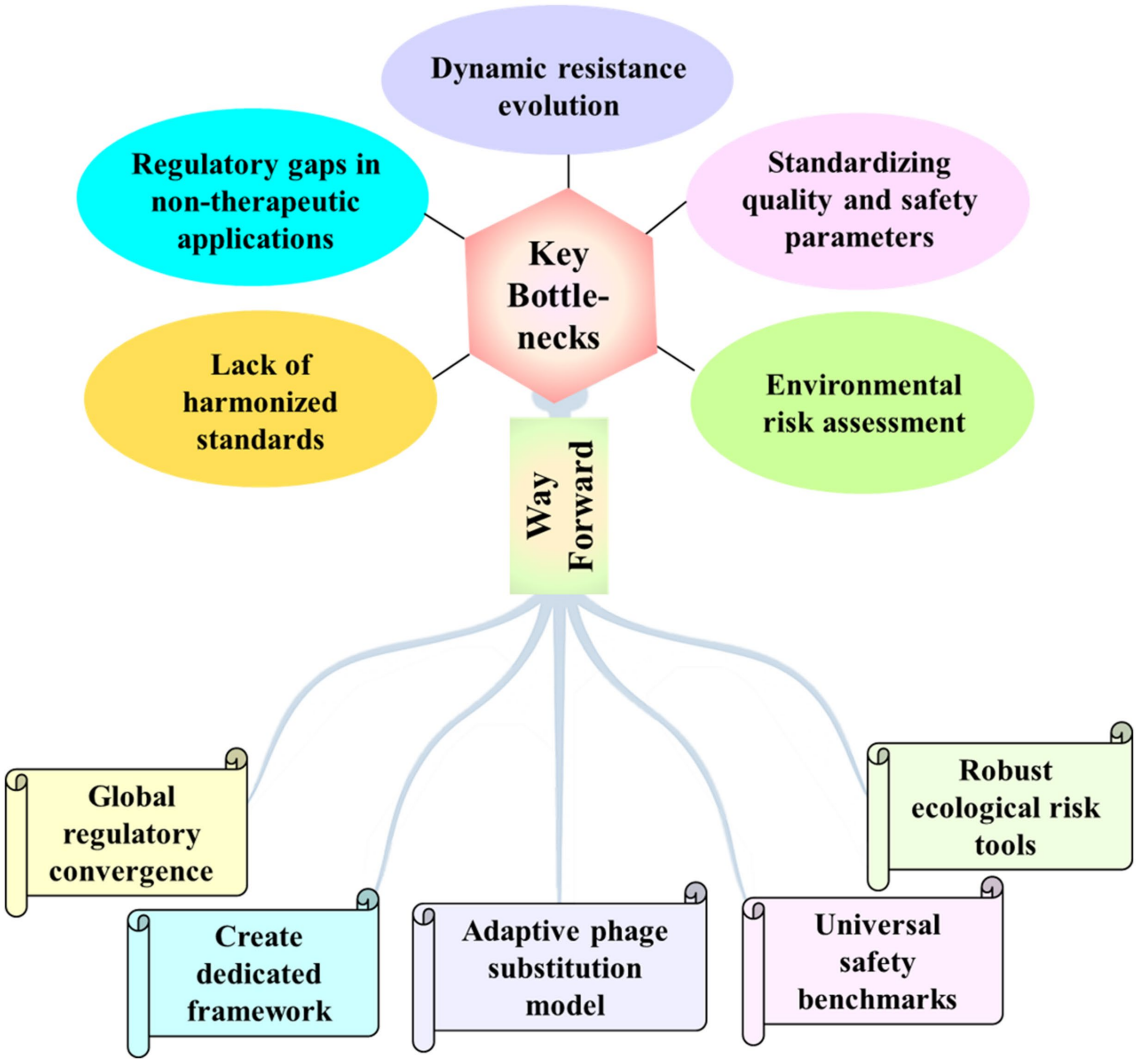
Schematic representation of key regulatory bottlenecks and strategic pathways to advance environmental phage applications.

In contrast to medicinal use of phages, regulation for environmental phage applications remains fragmented and immature (Samson et al., 2024). The U. S. Environmental Protection Agency has approved a handful of agricultural sprays under its microbial-biopesticide pathway (EPA Biopesticide Registration Notices), yet there is still no defined route for water disinfection, corrosion control or biofuel fermenters, and the European Union relies on generic biocide or feed-additive law rather than a phage-specific framework. In practice, applicants everywhere are asked for the same core dossier, i.e., genomic safety, potency and identity over shelf-life for every constituent, and evidence that each phage earns its place, however, timelines and evidentiary depth vary widely. Harmonized guidance for non-clinical sectors, formal adoption of dynamic “phage-bank” licensing, and expanded use of real-time release testing (RTRT) would significantly streamline regulatory approval for environmental phage products. Ongoing EPA biopesticide approvals and EMA draft guidance/concept papers provide a framework, but a dedicated environmental-phage regulatory pathway, ideally harmonized under a One-Health umbrella—will be crucial to move from lab to routine practice (Figure 1).

## 5 Future direction and concluding remarks

This minireview highlights bacteriophages as a precise, scalable, and promising solution for microbial threats across soil, bioenergy, and water environmental systems, addressing urgent challenges posed by antimicrobial resistance within One-Health framework. Despite compelling proof-of-concept studies and increasing patent activity concentrated in China and the United States, a clear gap persists between laboratory innovation and commercial deployment. To bridge this divide, strategic prioritization of high-margin sectors with clear unmet needs is essential for the commercial viability of environmental phage technologies, especially in light of ongoing challenges in production scalability, regulatory complexity, and competition from established chemical controls. Key targets include high-value specialty agricultural systems, MIC control in critical pipeline infrastructure, contamination management in specialty fermentations, and outbreak interventions in complex water systems.

For future development, addressing host-specificity will require advances in genomics-guided cocktail design, machine-learning host prediction, and receptor engineering to broaden coverage while preserving microbial community integrity. Although scalable and cost-effective GMP manufacturing is advancing, the regulatory frameworks for environmental phage applications remain fragmented. Hence, harmonization of guidelines, formal adoption of dynamic “phage-bank” licensing, and dedicated regulatory pathways, will be essential for facilitating market access. Collectively, these efforts position environmental phage technologies as a vital, sustainable tool for combating antimicrobial resistance at its source, offering transformative potential for agriculture, bioenergy and water systems.
